# Impact of double J stenting or nephrostomy placement during transurethral resection of bladder tumour on the incidence of metachronous upper urinary tract urothelial cancer

**DOI:** 10.1186/s12885-020-6620-2

**Published:** 2020-02-21

**Authors:** Marie C. Hupe, Lukas Dormayer, Tomasz Ozimek, Julian P. Struck, Martin J. P. Hennig, Melanie Klee, Christoph A. J. von Klot, Markus A. Kuczyk, Axel S. Merseburger, Mario W. Kramer

**Affiliations:** 1 Department of Urology, University Hospital Schleswig-Holstein, Campus Luebeck, Ratzeburger Allee 160, 23538 Luebeck, Germany; 2 Department of Urology, Medical School Hannover, Carl-Neuberg-Strasse 1, 30265 Hannover, Germany

**Keywords:** Upper urinary tract urothelial cancer, Double J stent, Nephrostomy, Transurethral resection of bladder tumour

## Abstract

**Background:**

Whether or not double J (DJ) stenting during transurethral resection of a bladder tumour (TURBT) harms patients with regard to possible metachronous upper urinary tract urothelial cancer (UUTUC) development remains controversial. This study evaluated the impact of DJ compared to nephrostomy placement during TURBT for bladder cancer (BCa) on the incidence of metachronous UUTUCs.

**Methods:**

We retrospectively analysed 637 patients who underwent TURBT in our department between 2008 and 2016. BCa, UUTUC and urinary drainage data (retrograde/anterograde DJ and percutaneous nephrostomy) were assessed, along with the prevalence of hydronephrosis, and mortality. Chi-square and Fisher’s exact test was performed for univariate analyses. Survival analysis was performed by the Kaplan-Meier method and log-rank tests.

**Results:**

UUTUC was noted in 28 out of 637 patients (4.4%), whereas only eight (1.3%) developed it metachronously to BCa. Out of these, four patients received DJ stents, while four patients received no urinary drainage of the upper urinary tract. Placement of urinary drainage significantly correlated with UUTUC (50.0% vs. 17.9%; *p* = 0.041). DJ stenting significantly correlated with UUTUC (50.0% vs. 11%; *p* <  0.01), while no patient with a nephrostomy tube developed UUTUC. UUTUC-free survival rates were significantly lower for patients with DJ stents than for all other patients (*p* = 0.001). Patients with or without DJ stents had similar overall survival (OS) rates (*p* = 0.73), whereas patients with nephrostomy tubes had significantly lower OS rates than all other patients (*p* <  0.001).

**Conclusions:**

Patients with DJ stenting during TURBT for BCa might have an increased risk of developing metachronous UUTUC. This study indicated advantages in placing nephrostomy tubes rather than DJ stents; however, confirmation requires investigation of a larger cohort. Even so, the increased mortality rate in the nephrostomy group reflected hydronephrosis as an unfavourable prognostic factor.

## Background

The majority of urothelial cancers (UC) are located in the bladder (> 90%), while < 10% are upper urinary tract urothelial cancers (UUTUC) [1]. In 17% of UUTUC patients synchronous bladder cancer (BCa) can also be found [1, 2]. UC recurs in the bladder during follow-up in up to 50% of all UUTUC patients, i.e., as BCa [1, 3]. By contrast, only 1.8% of patients with non-muscle invasive BCa (NMIBC) show synchronous UUTUC [4], and post-cystectomy upper urinary tract recurrences occur in < 10% of BCa patients [5–10]. Risk factors for post-cystectomy UUTUC development include history of carcinoma in situ (CIS) or recurrent BCa, cystectomy for NMIBC, and tumour involvement of the distal ureter or prostatic urethra [7, 8, 10]. In addition, risk factors for metachronous UUTUC following BCa diagnosis include high grade BCa and tumour localisation at the trigone/ureteral orifice [11, 12].

At time of initial diagnosis, 7.5 and 2.1% of all NMIBC patients present with unilateral and bilateral hydronephrosis, respectively. Among radical cystectomy patients unilateral and bilateral hydronephrosis is present in 19 and 4%, respectively [9]. Hydronephrosis is known to be associated with advanced BCa, unfavourable survival, as well as recurrence and progression [13–15]. While a nephrostomy tube is placed only in cases of hydronephrosis for drainage, a DJ stent can be placed under the same scenario, but also to protect the ureteral orifice during transurethral resection of a bladder tumour (TURBT), preventing vesicoureteral obstruction.

Kiss et al. retrospectively analysed radical cystectomy patients and indicated a higher risk for metachronous UUTUC in patients who underwent DJ stenting prior to radical cystectomy, compared to a nephrostomy tube or no urinary drainage of the upper urinary tract. Consequently, the authors recommended nephrostomies as preoperative drainage [9].

There are two main hypotheses for an increased UUTUC rate subsequent to DJ stenting during TURBT, enabling tumour cell seeding in the upper urinary tract: (I) a reflux volume between bladder and upper urinary tract, and (II) retrograde manipulation during DJ placement [9]. This study evaluated the impact of placement of a DJ stent compared to a nephrostomy tube during TURBT for BCa on the incidence of metachronous UUTUC.

## Methods

### Cohort

**Fig. 1:**
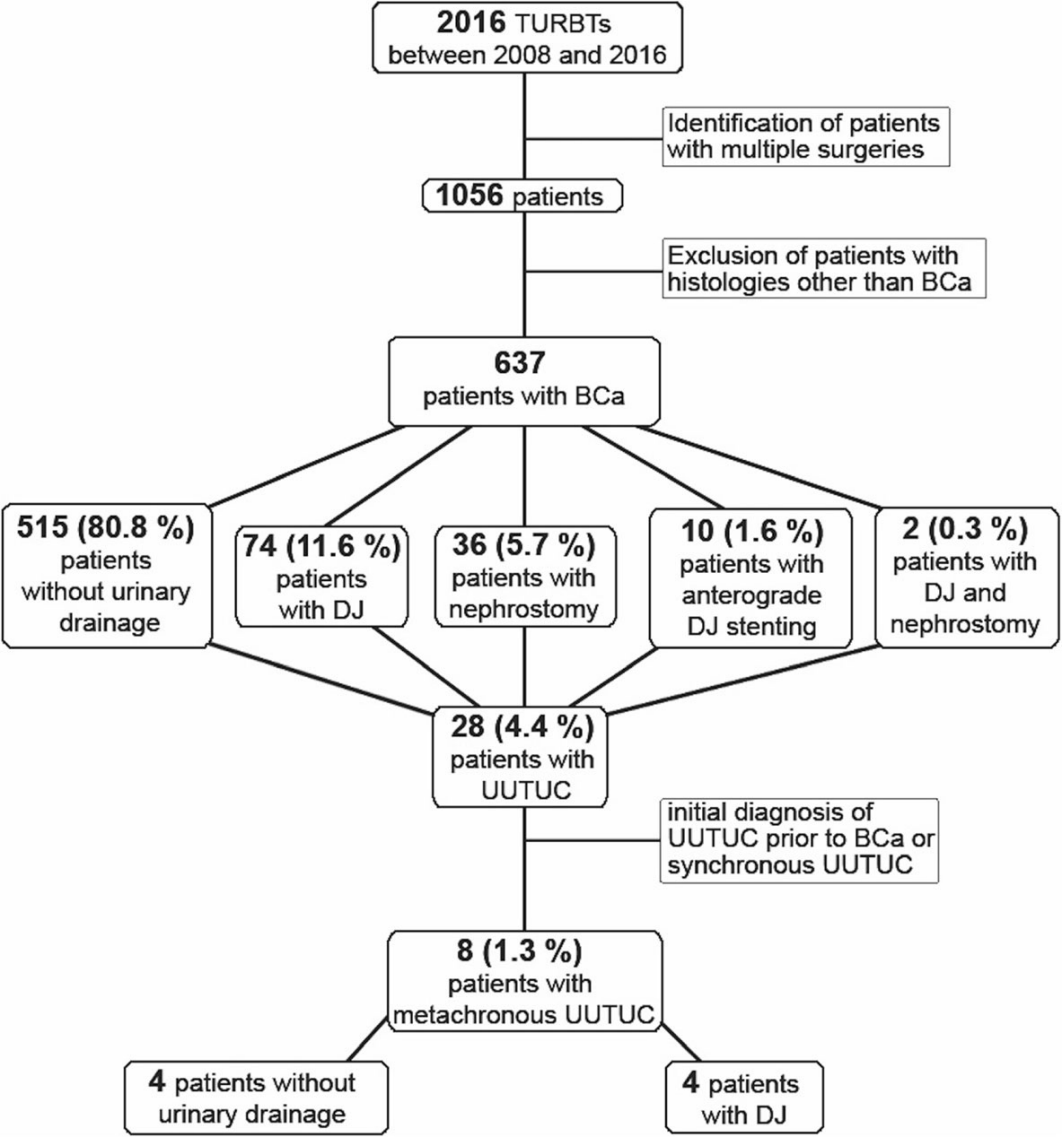
Flowchart of patient selection

Figure 1 gives an overview of patient selection for our cohort. Between 2008 and 2016, 2016 TURBTs were performed on 1056 patients at the Department of Urology, University Hospital Schleswig-Holstein (UKSH), Luebeck, Germany. Histologies other than BCa, (such as benign histologies, squamous cell carcinoma, adenocarcima; *n* = 419 patients) were excluded from the analysis. Variants of urothelial cancers were included. As a result, 637 patients were retrospectively analysed. Ethical approval was obtained from the local ethics committee at the University of Luebeck (17-354A9).

### Data collection

**Fig. 2:**
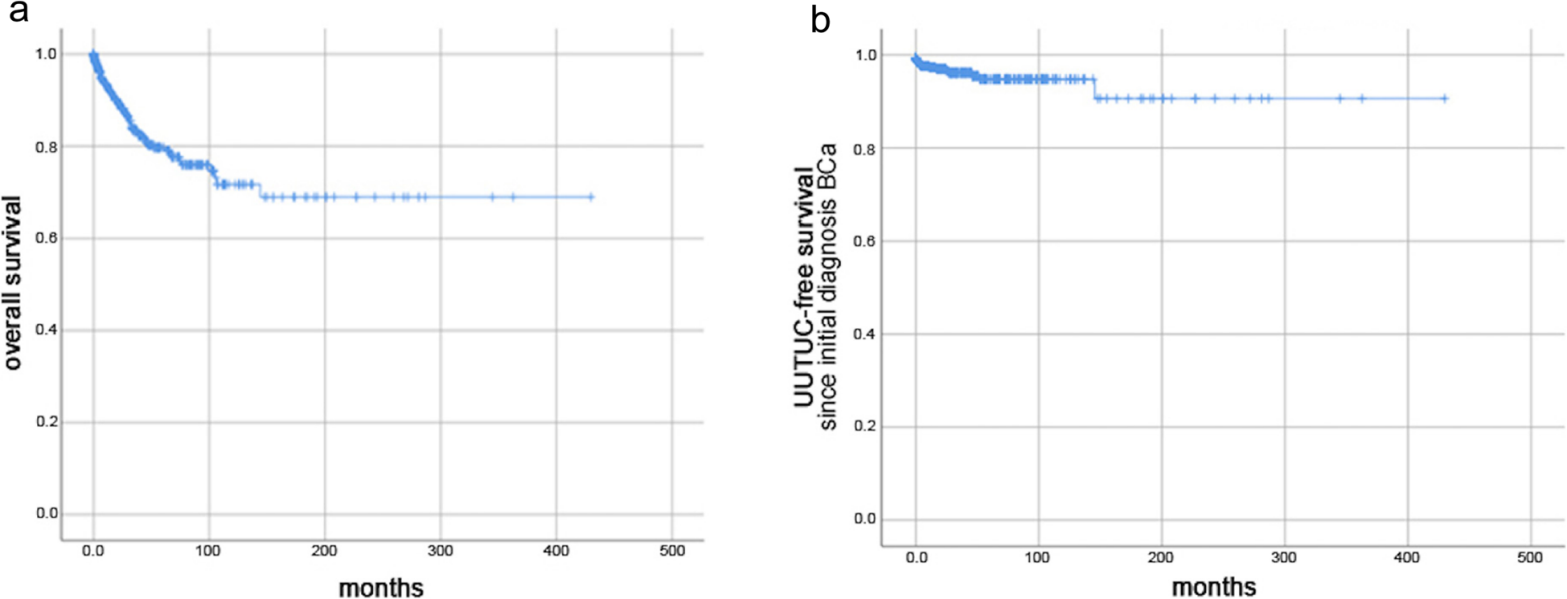
Survival data of entire cohort. **a** Overall survival of entire cohort (*n* = 637 patients; 77 events) (**b**) UUTUC-free survival since initial diagnosis of BCa for entire cohort (*n* = 617 patients; eight events)

**Fig. 3:**
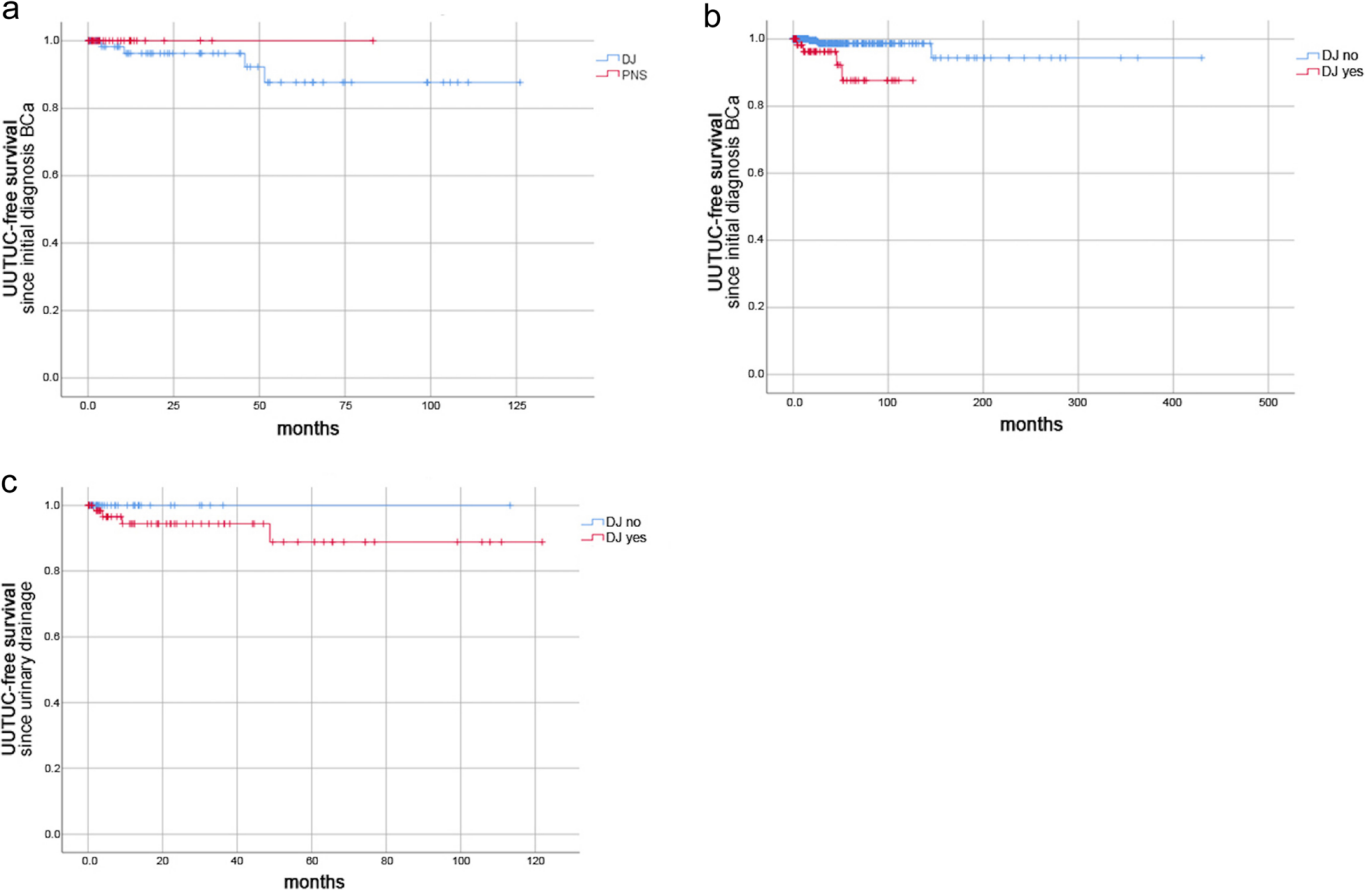
UUTUC-free survival data according to urinary drainage of the upper urinary tract. **a** UUTUC-free survival since initial diagnosis BCa for patients with DJ stents compared to those with nephrostomy tubes (*n* = 103 patients; four events; *p* = 0.415) (**b**) UUTUC-free survival since initial diagnosis BCa for patients with DJ stents compared to those without DJ stents (*n* = 617; eight events; *p* = 0.001) (**c**) UUTUC-free survival rates since urinary drainage of the upper urinary tract for patients with DJ stents compared to those without DJ stents (*n* = 113; four events; *p* = 0.26)

**Fig. 4:**
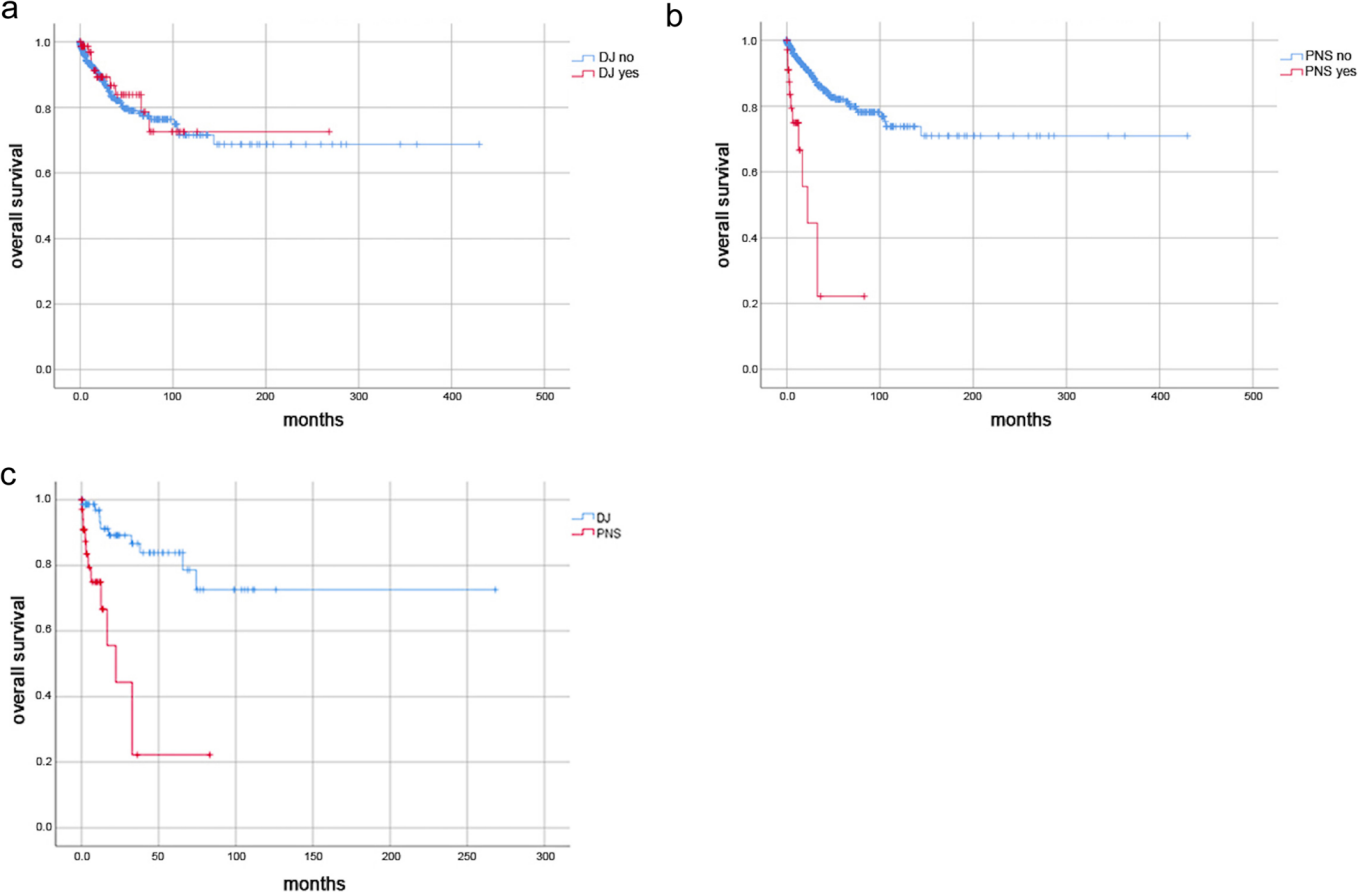
Overall survival data according to urinary drainage of the upper urinary tract. **a** Overall survival of entire cohort for patients with DJ stents compared to those without DJ stent (*n* = 637; 77 events; *p* = 0.73) (**b**) Overall survival of entire cohort for patients with nephrostomy tubes compared to those without nephrostomy tubes (*n* = 637; 77 events; *p* <  0.001) (**c**) Overall survival of entire cohort for patients with DJ stents compared to those with nephrostomy tubes (*n* = 110; 22 events; *p* <  0.001)

The following parameters were assessed: date of birth; gender; BCa grading/staging; UUTUC grading/staging/localisation; type of urinary drainage at time of TURBT (retrograde/anterograde DJ, percutaneous nephrostomy) including localisation; and the presence of hydronephrosis including localisation. Date of the last follow-up or death was used for follow-up and Kaplan-Meier analysis. Our analysis included patients who received their initial diagnosis of BCa prior to 2008, resulting in long follow-up periods (up to 400 months in Kaplan-Meier curves; Figs. 2, 3 and 4). The oldest diagnosis of BCa was in June 1979. In cases of missing World Health Organization (WHO) 2004 grading, G1 tumours were assigned a low-grade, G3 a high-grade and G2 an unknown grade. Data collection was completed in February 2018. “Metachronous UUTUC” was defined as an UUTUC that developed > 3 months from diagnosis of BCa and was diagnosed either by clear radiological evidence or by biopsy.

### Statistics

Statistical analysis was performed with SPSS Statistics for Windows, version 25.0 (IBM Corp., Armonk, NY, USA). For descriptive data, we determined the median and mean with standard deviation. Chi-square analysis and Fisher’s exact test (event rates < 10) were performed for categorical variables. Survival analysis was performed by the Kaplan-Meier method and log-rank tests. Significance was defined as *p* <  0.05. UUTUC-free survival was defined as the period between initial diagnosis of BCa, or time of urinary drainage of the upper urinary tract, and either diagnosis of UUTUC, last follow-up or death. Overall survival (OS) was defined as the period between initial diagnosis of BCa and either last follow-up or death. Patients who developed UUTUC prior to or synchronous with BCa (*n* = 20) were excluded from all UUTUC-free survival analyses.

### Data accessibility

The data supporting the findings of this study are available on request from the corresponding author. The data are not publicly available due to privacy and ethical restrictions.

## Results

### Patient characteristics

Patient characteristics are shown in Table 1. The majority of patients presented with NMIBC at initial diagnosis (pTa/pT1/pTis: 72.3%). Concomitant CIS at initial diagnosis was present in 7.5% of patients. Only 13.3% of the patients presented with hydronephrosis. Urinary drainage (DJ or percutaneous nephrostomy) was initiated in 19.2% of patients. In total, 28 out of 637 patients (4.4%) suffered from UUTUC, whereas only eight patients with UUTUC (1.3%) developed it during BCa follow-up (i.e. metachronously). Out of these eight patients, four received a DJ stent while the other four received no urinary drainage of the upper urinary tract. Figure 2a shows the OS in our cohort. Figure 2b shows the UUTUC-free survival of our cohort. The median follow-up of our cohort was 14.9 months from initial BCa diagnosis and 12 months from urinary drainage of the upper urinary tract. At the end of follow-up the mortality rate in the overall cohort was 12.1% (77/637). Table 2 presents further characteristics of the 17 patients with synchronous or metachronous UUTUC, and Table 3 displays pathological data for the eight patients with metachronous UUTUC.

**Table 1:** Patient characteristics

Parameter	Total *n* = 637
Gender	
male	79.4% (506/637)
female	20.6% (131/637)
Age at initial diagnosis BCa (mean ± SD; years)	72.5 ± 11.5
T stage BCa at initial diagnosis	
pTa	41.8% (266/637)
pT1	30.0% (191/637)
pT2	26.7% (170/637)
pT3	0.3% (2/637)
pT4	0.2% (1/637)
pTis	0.5% (3/637)
PUNLMP	0.5% (3/637)
unknown	0.2% (1/637)
Concomitant CIS at initial diagnosis	
yes	7.5% (48/637)
no	92.5% (589/637)
Grading (WHO 1973) BCa at initial diagnosis	
G1	18.7% (119/637)
G2	44.4% (283/637)
G3	36.6% (233/637)
unknown	0.3% (2/637)
Grading (WHO 2004) BCa at initial diagnosis	
low-grade	20.4% (130/637)
high-grade	74.9% (477/637)
unknown	4.7% (30/637)
Hydronephrosis	
no	86.8% (553/637)
left	3.8% (24/637)
right	4.6% (29/637)
bilateral	4.9% (31/637)
Urinary drainage of the upper urinary tract	
no	80.8% (515/637)
DJ	11.6% (74/637)
percutaneous nephrostomy	5.7% (36/637)
percutaneous nephrostomy for anterograde DJ	1.6% (10/637)
percutaneous nephrostomy and DJ	0.3% (2/637)
Upper urinary tract urothelial carcinoma (UUTUC)	
no	95.6% (609/637)
yes	4.4% (28/637)
UUTUC prior to BCa	39.3% (11/28)
UUTUC synchronous to BCa	32.1% (9/28)
UUTUC metachronous to BCa	28.6% (8/28)
Death	
no	87.9% (560/637)
yes	12.1% (77/637)

*BCa* bladder cancer, *CIS* carcinoma in situ, *DJ* double J stent, *PUNLMP* papillary urothelial neoplasm of low malignant potential, *SD* standard deviation, *UUTUC* upper urinary tract urothelial cancer, *WHO* World Health Organization

**Table 2:** Characteristics of all 17 patients with synchronous or metachronous UUTUC

Patient #	Gender	Hydronephrosis	Urinary drainage of the upper urinary tract	Time from diagnosis BCa to UUTUC (months)	Localisation UUTUC	T-stage and grading UUTUC	synchronous (s) vs. metachronous (m) UUTUC^a^
1	female	bilateral	nephrostomy bilateral	0.4	left	pT4, G2, high-grade	s
2	male	left	anterograde DJ left	2.1	left	pTis, G3	s
3	male	no	no	n.a.	right	pT1, G2, n.a.	n.a.
4	male	left	DJ left	45.8	left	pTa, G2, high-grade	m
5	male	bilateral	DJ bilateral	10.6	left	pTa, G1	m
6	male	right	nephrostomy right	0.2	right	pT1, G3	s
7	male	no	no	26.9	right	pT2, G3	m
8	male	left	no	16.2	left	pT2, G2, n.a.	m
9	male	no	DJ bilateral	3.9	right	pT1, G2, high-grade	m
10	female	left	DJ left	0.2	left	pT3, G3	s
11	male	no	no	24.9	left	pTa, G1	m
12	female	right	DJ right	0.3	right	pTa, G2, high-grade	s
13	male	no	no	145.5	left	pTa, G1	m
14	male	right	nephrostomy right	1.2	right	pTis, G3	s
15	male	right	DJ right	1.5	right	pTa, G2, n.a.	s
16	male	right	DJ right	51.5	right	pT2, G2, high-grade	m
17	male	right	no	2.8	right	pT2, G2, n.a.	s

^a^ Synchronous UUTUC < 3 months from diagnosis of BCa, metachronous > 3 months from diagnosis of BCa; *BCa* bladder cancer, *DJ* double J stent, *n.a.* not assessable, *UUTUC* upper urinary tract urothelial cancer

**Table 3:** Pathological data of the eight patients with metachronous UUTUC

UUTUC metachronous to BCa	*n* = 8
T-stage UUTUC	
pTa	37.5% (3/8)
pT1	37.5% (3/8)
pT2	25.0% (2/8)
Grading (WHO 1973) UUTUC	
G1	25.0% (2/8)
G2	50.0% (4/8)
G3	25.0% (2/8)
Grading (WHO 2004) UUTUC	
low-grade	25.0% (2/8)
high-grade	62.5% (5/8)
unknown	12.5% (1/8)

*BCa* bladder cancer, *UUTUC* upper urinary tract urothelial cancer, *WHO* World Health Organization

All four patients with UUTUC and DJ developed UUTUC at the same location (left/right) as the DJ stent was placed (Table 2; patients #4, 5, 9, and 16). Out of these, one patient without hydronephrosis received bilateral DJ stents, i.e., as a protective measure (patient #9). Median time from stent placement to UUTUC development for these four patients was 28.2 months.

### Parameters correlating with UUTUC

Presence of hydronephrosis did not significantly correlate with metachronous UUTUC (Table 4). In general, placement of urinary drainage of the upper urinary tract significantly correlated with UUTUC (50.0% vs. 17.9%; *p* = 0.041). No patient with UUTUC had a nephrostomy. DJ stenting significantly correlated with UUTUC (50.0% vs. 11%; *p* <  0.01). Grading and staging at time of initial BCa diagnosis, or at time of urinary drainage of the upper urinary tract, did not correlate with UUTUC.

**Table 4:** Parameters correlating with occurrence of UUTUC

Parameter	No metachronous UUTUC	Metachronous UUTUC present	*p*-value (^§^ Chi-square; ^#^ Fisher’s exact)
Hydronephrosis			**n.s.**
no	87.7% (534/609)	62.5% (5/8)	
yes	12.3% (75/609)	37.5% (3/8)	
Urinary drainage of the upper urinary tract			**0.041** ^**#**^
no	82.1% (500/609)	50.0% (4/8)	
yes	17.9% (109/609)	50.0% (4/8)	
Type of urinary drainage			**n.s.**
DJ	65.7% (65/99)	100% (4/4)	
nephrostomy	34.3% (34/99)	0% (0/4)	
DJ			**0.007** ^**#**^
no (incl. nephrostomy)	89.3% (544/609)	50.0% (4/8)	
yes	10.7% (65/609)	50.0% (4/8)	
DJ			**0.009** ^**#**^
no (excl. nephrostomy)	88.7% (510/575)	50.0% (4/8)	
yes	11.3% (65/575)	50.0% (4/8)	
Nephrostomy			**n.s.**
no (incl. DJ)	94.4% (575/609)	100.0% (8/8)	
yes	5.6% (34/609)	0% (0/8)	
Nephrostomy			**n.s.**
no (excl. DJ)	93.8% (510/544)	100% (4/4)	
yes	6.3% (34/544)	0% (0/4)	
T-stage BCa at initial diagnosis			**n.s.**
pTa	42.1% (253/601)	62.5% (5/8)	
pT1	30.3% (182/601)	37.5% (3/8)	
pT2	27.6% (166/601)	0% (0/8)	
miscellaneous	1.2% (8/609)	0% (0/8)	
Grading (WHO 1973) BCa at initial diagnosis			**n.s.**
G1	18.0% (109/607)	50.0% (4/8)	
G2	45.1% (274/607)	25.0% (2/8)	
G3	36.9% (224/607)	25.0% (2/8)	
unknown	0.3% (2/609)	0% (0/8)	
Grading (WHO 2004) BCa at initial diagnosis			**n.s.**
low-grade	20.7% (120/581)	50.0% (4/8)	
high-grade	79.3% (461/581)	50.0% (4/8)	
unknown	4.6% (28/609)	0% (0/8)	
T-stage BCa at urinary drainage of the upper urinary tract			**n.s.**
pTa	29.5% (31/105)	75.0% (3/4)	
pT1	17.1% (18/105)	25.0% (1/4)	
pT2	53.3% (56/105)	0% (0/4)	
miscellaneous	3.7% (4/109)	0% (0/4)	
Grading (WHO 1973) BCa at urinary drainage of the upper urinary tract			**n.s.**
G1	10.3% (11/107)	25.0% (1/4)	
G2	41.1% (44/107)	50.0% (2/4)	
G3	48.6% (52/107)	25.0% (1/4)	
unknown	1.8% (2/109)	0% (0/4)	
Grading (WHO 2004) BCa at urinary drainage of the upper urinary tract			**n.s.**
low-grade	10.4% (11/106)	25.0% (1/4)	
high-grade	89.6% (95/106)	75.0% (3/4)	
unknown	2.8% (3/109)	0% (0/4)	

Data excludes synchronous UUTUCs; *n* = 617; miscellaneous includes BCa staging >pT2, pTis and PUNLMP; “miscellaneous” and “unknown” categories were excluded from statistical analyses

*BCa* bladder cancer, *DJ* double J stent, *n.s.* not significant, *UUTUC* upper urinary tract urothelial cancer, *WHO* World Health Organization

UUTUC-free survival rates since initial diagnosis of BCa were lower for patients with DJ stents than for those with nephrostomy tubes, with no events being recorded for the nephrostomy group (Fig. 3a; *p* = 0.415). Compared to all other patients, UUTUC-free survival rates since initial diagnosis BCa for patients with DJ stents were significantly lower (Fig. 3b; *p* = 0.001). UUTUC-free survival rates since time of urinary drainage of the upper urinary tract were lower for patients with DJ stents, compared to the remaining patients (Fig. 3c; *p* = 0.26). All metachronous UUTUCs in the group with DJ stents occurred within 5 years of drainage placement.

### Parameters correlating with mortality

Presence of hydronephrosis, and urinary drainage of the upper urinary tract in general, significantly correlated with death (both *p* <  0.0001; Table 5). However, while nephrostomy tubes as urinary drainage significantly correlated with death (*p* <  0.0001), this was not the case for DJ stenting. Advanced T-stage and grading at initial diagnosis also significantly correlated with death (*p* <  0.0001 and *p* <  0.03). UUTUC (prior to BCa, synchronous or metachronous) did not correlate with death. From Kaplan-Meier analysis, patients with or without DJ stents had similar OS rates (Fig. 4a; *p* = 0.73), whereas patients with nephrostomy tubes had significantly lower OS rates than the other patients (Fig. 4b; *p* <  0.001). Patients with nephrostomy tubes died within 5 years of the initial diagnosis of BCa. Patients with nephrostomy tubes also had significantly lower OS rates when exclusively compared to those with DJ stents (Fig. 4c; *p* <  0.001).

**Table 5:** Parameters correlating with mortality

Parameter	Patient survived	Patient died	*p*-value (^§^ Chi-square; ^#^ Fisher’s exact)
Hydronephrosis			**< 0.0001** ^**§**^
no	89.1% (499/560)	70.1% (54/77)	
yes	10.9% (61/560)	29.9% (23/77)	
Urinary drainage of the upper urinary tract			**< 0.0001** ^**§**^
no	83.0% (465/560)	64.9% (50/77)	
yes	17.0% (95/560)	35.1% (27/77)	
Type of urinary drainage			**0.015** ^**§**^
DJ	72.7% (64/88)	45.5% (10/22)	
nephrostomy	27.3% (24/88)	54.5% (12/22)	
DJ (incl. nephrostomy)			**n.s.**
no	88.6% (496/560)	87.0% (67/77)	
yes	11.4% (64/560)	13.0% (10/77)	
DJ (excl. nephrostomy)			**n.s.**
no	88.1% (472/536)	84.6% (55/65)	
yes	11.9% (64/536)	15.4% (10/65)	
Nephrostomy (incl. DJ)			**< 0.0001** ^**§**^
no	95.7% (536/560)	84.4% (65/77)	
yes	4.3% (24/560)	15.6% (12/77)	
Nephrostomy (excl. DJ)			**< 0.0001** ^**§**^
no	95.2% (472/496)	82.1% (55/67)	
yes	4.8% (24/496)	17.9% (12/67)	
T-stage BCa at initial diagnosis			**< 0.0001** ^**§**^
pTa	45.9% (253/551)	17.1% (13/76)	
pT1	28.7% (158/551)	43.4% (33/76)	
pT2	25.4% (140/551)	39.5% (30/76)	
miscellaneous	1.6% (9/560)	1.3% (1/77)	
Grading (WHO 1973) BCa at initial diagnosis			**< 0.0001 §** **(G1 vs. G3 < 0.0001**^**#**^**; G1 vs. G2 0.027**^**#**^**; G2 vs. G3 0.005**^**#**^**)**
G1	20.6% (115/559)	5.3% (4/76)	
G2	45.6% (255/559)	36.8% (28/76)	
G3	33.8% (189/559)	57.9% (44/76)	
unknown	0.2% (1/560)	1.3% (1/77)	
Grading (WHO 2004) BCa at initial diagnosis			**< 0.0001** ^**#**^
low-grade	23.8% (126/530)	5.2% (4/77)	
high-grade	76.2% (404/530)	94.8% (73/77)	
unknown	5.4% (30/560)	0% (0/77)	
UUTUC			**n.s.**
no	95.5% (535/560)	96.1% (74/77)	
yes	4.5% (25/560)	3.9% (3/77)	

Data includes synchronous UUTUCs; *n* = 637; miscellaneous includes BCa staging >pT2, pTis and PUNLMP; “miscellaneous” and “unknown” categories were excluded from statistical analyses)

*BCa* bladder cancer, *DJ* double J stent, *n.s.* not significant, *UUTUC* upper urinary tract urothelial cancer, *WHO* World Health Organization

### Parameters correlating with hydronephrosis

Advanced T-staging and grading of BCa at initial diagnosis significantly correlated with hydronephrosis (*p* <  0.0001, *p* <  0.012 and *p* <  0.033, respectively; Table 6). All nephrostomies were placed in cases of hydronephrosis; however, only half of the DJ stents were placed in cases of hydronephrosis (Table 6).

**Table 6:** Parameters correlating with hydronephrosis

Parameter	Hydronephrosis absent	Hydronephrosis present	*p*-value (^§^ Chi-square; ^#^ Fisher’s exact)
T-stage BCa at initial diagnosis			**< 0.0001** ^**§**^
pTa	45.7% (250/547)	20.0% (16/80)	
pT1	31.6% (173/547)	22.5% (18/80)	
pT2	22.7% (124/547)	57.5% (46/80)	
miscellaneous	1.1% (6/553)	4.8% (4/84)	
Grading (WHO 1973) BCa at initial diagnosis			**0.012** ^**§**^
G1	19.7% (109/553)	12.2% (10/82)	
G2	45.8% (253/553)	36.6% (30/82)	
G3	34.5% (191/553)	51.2% (42/82)	
unknown	0% (0/553)	2.4% (2/84)	
Grading (WHO 2004) BCa at initial diagnosis			**0.033** ^**§**^
low-grade	22.8% (120/526)	12.3% (10/81)	
high-grade	77.2% (406/526)	87.7% (71/81)	
unknown	4.9% (27/553)	3.6% (3/84)	
Type of urinary drainage			**< 0.0001** ^**#**^
DJ	100% (38/38)	50.0% (36/72)	
nephrostomy	0% (0/38)	50.0% (36/72)	

Data includes synchronous UUTUCs; *n* = 637 (*n* = 110 for urinary drainage of the upper urinary tract analysis); miscellaneous includes BCa staging >pT2, pTis and PUNLMP; “miscellaneous” and “unknown” categories were excluded from statistical analyses

*BCa* bladder cancer, *WHO* World Health Organization

## Discussion

There is a debate as to whether DJ stenting during TURBT harms patients with regard to possible metachronous UUTUC development. Why is this? On the one hand, there is the aforementioned assumption of UUTUC caused by DJ implementation via retrograde tumour cell seeding. On the other hand, not every UUTUC results in synchronous or metachronous BCa although there is a constant tumour cell seeding from the upper to the lower urinary tract.

The main findings of our study were: (I) patients with DJ stenting during TURBT for BCa were at increased risk for UUTUC development during follow-up, compared to patients with nephrostomies or no urinary drainage of the upper urinary tract during TURBT; (II) an increased risk of mortality in cases of nephrostomy placement for urinary drainage; and (III) a notably low incidence of metachronous UUTUC in general (1.3%).

All patients with UUTUC and DJ stenting in our cohort developed UUTUC congruent with the stent location (Table 2). However, not every DJ stent at the time of TURBT resulted in a metachronous UUTUC. Despite the low UUTUC incidence, our results suggested that a nephrostomy tube should be placed in cases of hydronephrosis, rather than a DJ stent. The increased mortality rate in the nephrostomy group mirrored that of hydronephrosis as an unfavorable prognostic factor. However, due to the high mortality rate among nephrostomy patients, UUTUC development during longer follow-up might be anticipated. Kaplan-Meier curves showed that deaths in the nephrostomy group (Fig. 4c) and UUTUC in the DJ stenting group (Fig. 3a and b) occurred within a 5 year follow-up. Thus, it remained unclear whether the use of nephrostomy tubes during TURBT might cause UUTUC over a longer period (> 5 years).

There are other studies evaluating the need for, and harmful effects of, DJ stenting in BCa patients; however, the results are controversial. Notably, our study was the first to compare the potential harm caused by both DJ stents and nephrostomy tubes during TURBT for BCa with regard to metachronous UUTUC.

Kiss et al. also retrospectively assessed the risk of urinary drainage with either a DJ stent or nephrostomy tube for UC recurrence in the upper urinary tract [9]. Their study design is similar to ours, however there are two major differences. First, Kiss et al. analysed a radical cystectomy cohort (*n* = 1005; vs. TURBT cohort in our study). Second, they assessed the impact of preoperative urinary drainage (vs. intraoperative urinary drainage in our cohort, i.e. increased risk of tumour cell seeding due to cutting/resection of the tumour). In their cohort, preoperative hydronephrosis was present in 4% of the patients bilaterally and in 19% unilaterally. Half of the patients with hydronephrosis underwent preoperative urinary drainage with either a DJ stent (46%) or nephrostomy tube (54%). In total, there were 3% UUTUC recurrences, including 13% of patients with DJ stents, 0% from the nephrostomy group and 3% from the no urinary drainage group. As such, the authors identified preoperative DJ stenting, but not hydronephrosis, as an independent risk factor for metachronous UUTUC. Consistent with our results, UUTUC-free survival was shorter in the DJ stenting group, and OS was shorter in the nephrostomy group. Consequently, Kiss et al. proposed the use of nephrostomies for preoperative urinary drainage of the upper urinary tract, when necessary [9].

A study by Chou et al. revealed ureteral orifice localisation of BCa in 31 out of 572 (5.4%) patients who underwent TURBT [12], with metachronous UUTUC occurring in four (12.9%) of these patients. DJ stents were placed in six patients due to surgical damage of the ureteral orifice during the procedure; however, there were no metachronous UUTUCs or vesicoureteral obstruction during their follow-up. On the contrary, vesicoureteral obstruction developed in three (10%) patients without DJ stenting due to scar formation of the ureteral orifice [12].

Mano et al. also examined the outcome of ureteral orifice resection during TURBT (*n* = 84) [16]. Patients with preoperative hydronephrosis and DJ stenting during the procedure were excluded. Postoperative hydronephrosis was documented in 13% of patients, with it being due to vesicoureteral obstruction in only three patients (4%). Only one patient showed UUTUC recurrence [16].

Altok et al. also retrospectively investigated TURBTs for BCa including the ureteral orifice (*n* = 138) [17]. There was no DJ stenting in this cohort. Synchronous and metachronous UUTUC developed in 10.1 and 5.3% of these cases, respectively. Postoperative hydronephrosis occurred in 19.5% of the patients without preoperative hydronephrosis due to vesicoureteral reflux (47%), disease progression including the ureteral orifice (29%), urolithiasis (3%), and vesicoureteral obstruction (6%; *n* = 1). Therefore, the authors recommended against routine DJ stenting during TURBT of the ureteral orifice [17].

Taken together and based in the aforementioned studies [12, 16, 17], the rate of postoperative vesicoureteral obstruction rate due to a TURBT close to the ureteral orifice seems to be low and thus DJ stenting to protect the ureteral orifice eventually abdicable. However, these studies include only small cohorts. Larger cohorts are certainly needed to support this assumption.

Limitations of our study were its retrospective design, including the lack of information about the cause of death; therefore, cancer specific mortality was not determined. There was no information about prior manipulation of the upper urinary tract for those BCa patients who were diagnosed prior to 2008 and about the DJ stent dwell time. Furthermore, tumour localization, number of tumours in the bladder as well as subsequent therapies or upper urinary tract manipulations were not investigated. There was no standardized screening for an UUTUC at the time of BCa diagnosis. Notably, the low incidence of metachronous UUTUCs in our cohort as well as the short median follow-up need to be considered during interpretation of the results. Thus, further investigation on larger cohorts and randomised studies comparing DJ with nephrostomy tube drainage during TURBT are needed to confirm these results.

## Conclusions

In conclusion, patients with DJ stenting during TURBT for BCa had an increased risk for UUTUC development during follow-up. The results indicated that a nephrostomy tube should be placed in cases of hydronephrosis, rather than DJ stent, if feasible. Previous reports demonstrated acceptably low rates of postoperative vesicoureteral obstruction during TURBT close to the ureteral orifice. Thus, DJ stenting to protect the ureteral orifice might be abdicable.

## Data Availability

The datasets generated during and analyzed during the current study are not publicly available due to privacy or ethical restrictions but are available from the corresponding author on reasonable request.

## Abbreviations

BCa: Bladder cancer
CIS: Carcinoma in situ
DJ: Double J stent
n.a: not assessable
n.s: not significant
NMIBC: Non-muscle invasive bladder cancer
OS: Overall survival
PUNLMP: Papillary urothelial neoplasm of low malignant potential
SD: Standard deviation
TURBT: Transurethral resection of a bladder tumour
UC: Urothelial cancers
UKSH: University Hospital Schleswig-Holstein
UUTUC: Upper urinary tract urothelial cancer
WHO: World Health Organization
